# Preliminary report of a gas conditioner to improve operational reliability of cryotherapy in developing countries

**DOI:** 10.1186/1472-6874-6-2

**Published:** 2006-02-06

**Authors:** Yancy Seamans, John Sellors, Fredrik Broekhuizen, Michelle Howard

**Affiliations:** 1 Program for Appropriate Technology in Health (PATH), Seattle, WA, USA; 2 Department of Obstetrics and Gynecology, Medical College of Wisconsin, Milwaukee, WI, USA; 3 Department of Family Medicine, McMaster University, Hamilton, Ontario, Canada

## Abstract

**Background:**

Cryotherapy is a safe, affordable, and effective method of treatment for cervical intraepithelial neoplasia. In some low-resource settings, environmental conditions or qualities of the refrigerant gas can lead to blockage of cryotherapy equipment, terminating treatment. A prototype of a gas conditioner to prevent operational failure was designed, built, and field tested.

**Methods:**

The prototype conditioner device consists of an expansion chamber that filters and dries the refrigerant gas. Users in Peru and Kenya reported on their experience with the prototype conditioner. In Ghana, simulated cryotherapy procedures were used to test the effects of the prototype conditioner, as well as the commonly used "cough technique."

**Results:**

Anecdotal reports from field use of the device were favorable. During simulated cryotherapy, the prevalence of blockage during freezing were 0% (0/25) with the device alone, 23.3% (7/30) with the cough technique alone, 5.9% (1/17) with both, and 55.2% (16/29) with neither (Pearson's Chi square = 26.6, df = 3, p < 0.001 (comparison amongst all groups)).

**Conclusion:**

This prototype design of a cryotherapy gas conditioner is a potential solution for low-resource settings that are experiencing cryotherapy device malfunction.

## Background

Cryotherapy to treat precancerous cervical lesions in low-resource settings has been shown to be both clinically effective and logistically possible [1-4]. However, experience in developing country clinics has indicated that cryotherapy units fail due to blockage (i.e. complete stoppage of the gas flow), and that preventive measures are advisable [5,6]. This problem arises when there is an interruption in gas flow during the procedure, and results in service disruption for several minutes while the unit thaws and the blockage clears. It can be argued that such interruptions might threaten the effectiveness of cryotherapy, since the procedure usually entails two 3-minute freezes, separated by a 5-minute interval to allow thawing of the tissue [7]. Typically, failures have been attributed to gas supply line blockages caused by gas impurities, condensation and freezing of water vapor, or formation of dry ice in the gas supply lines.

JHPIEGO currently advocates a "freeze-clear-freeze technique (also known as the "cough" technique) to alleviate blockages [5]. The cough technique involves briefly interrupting freezing every 15 seconds by depressing the defrost button for no more than one second and then resuming freezing. While potentially effective, the applicability of this technique is limited because it can only be used with a device incorporating an active defrost function (e.g., Wallach LL100, Wallach Surgical Devices, Orange, Connecticut) and cannot be used with other devices (e.g., Ascon Multi Tip Cryo MTC-810, Ascon Medical Instruments, Chennai, India) that do not have this function. This paper describes the design and construction of a prototype of an inexpensive gas conditioner device, and assesses its field performance in low-resource settings. In clinic settings in two countries using N_2_O and CO_2 _as the refrigerant gas, anecdotal reports of effectiveness were obtained from clinicians. In a third setting, evaluations were conducted on nonhuman tissue with CO_2_, comparing the prevalence of blockage with and without the conditioner device and use of the cough technique.

## Methods

### Device design and construction

The prototype device was designed to address the hypothesized reasons for cryotherapy device failure due to gas blockage: the presence of liquid refrigerant and subsequent formation of dry ice, excessive moisture in the gas leading to ice formation, and foreign matter. The device functions as an expansion chamber (converting any liquid refrigerant to gas), a gas dryer to remove excess moisture, and a large-pore filter to remove any large particulate contaminants.

**Figure 1:**
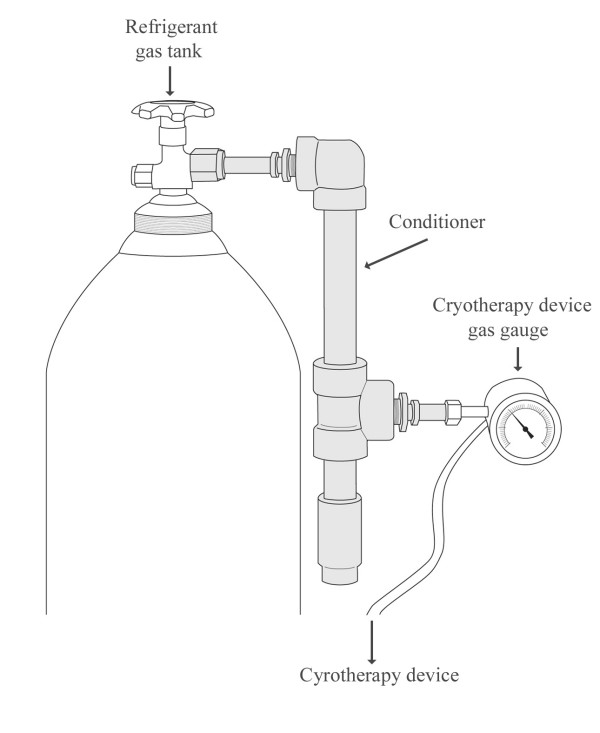
A sketch of the cryotherapy gas conditioner device. Sketch represents gas conditioner device attached to the tank of refrigerant gas with the cryotherapy unit connected to the other end of the gas conditioner.

**Figure 2:**
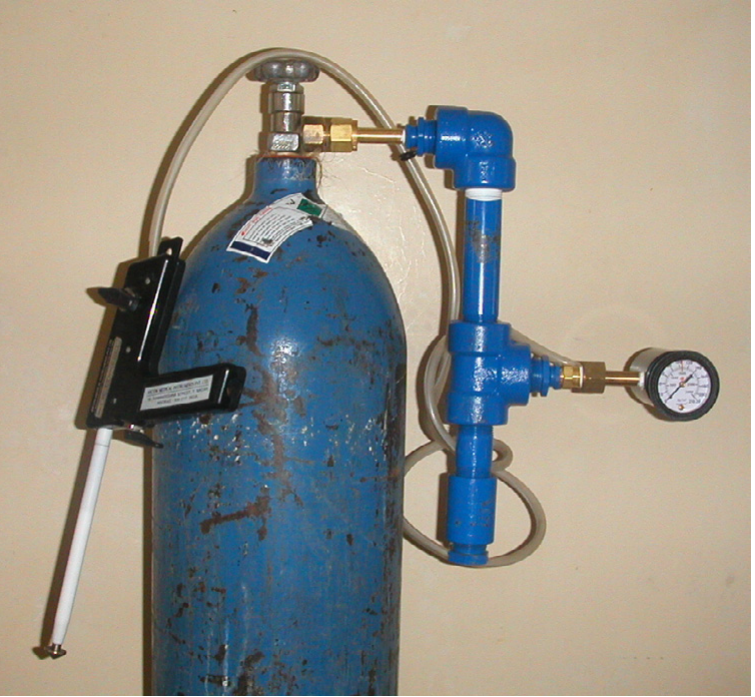
A picture of the cryotherapy gas conditioner with an Ascon Cryosurgery device.

**Figure 3:**
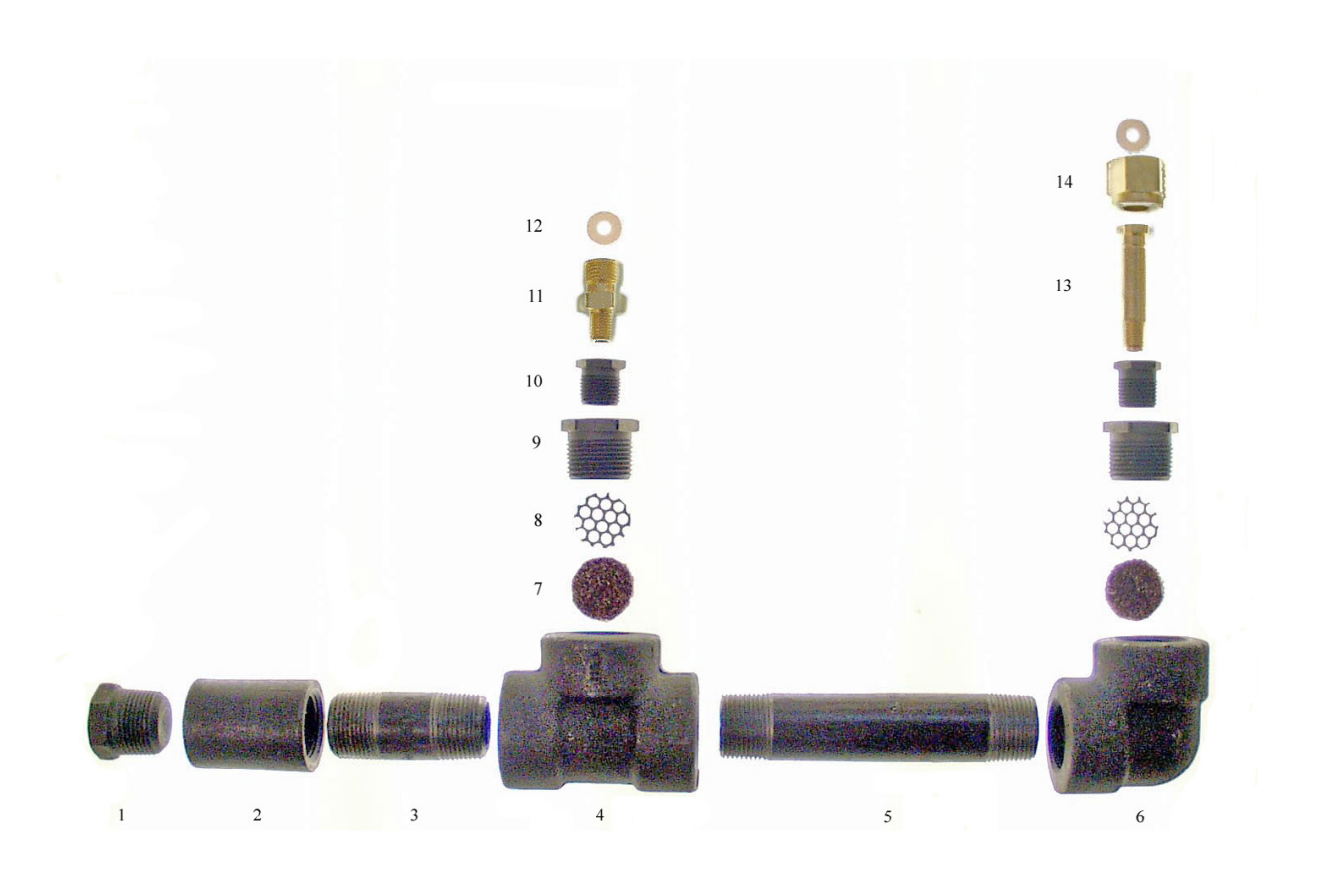
An exploded view of the cryotherapy gas conditioner assembly.

The conditioner (Figures 1 and 2) is constructed from off-the-shelf materials and requires minimal assembly. The main body is constructed from Schedule 80 pipe and fittings, which are rated at 3000 pounds per square inch (psi) to ensure an engineering design factor of three to four for safety (tanks typically provide gas at 750 – 1000 psi). The prototype conditioner was constructed with gas fittings appropriate for US N_2_O or CO_2 _gas cylinders (CGA 326 or 320) but can be designed with fittings appropriate for the country in which the device is used. The desiccating material used in the conditioner is Drierite (W.A. Hammond Drierite Co. Ltd., Xenia, Ohio), a commercially available calcium sulfate laboratory desiccant, commonly used to dry laboratory gases. This desiccant can be heated in a warm oven to restore its properties and allow for extended use. The weight of an assembled device is 4.4 kg and measures 42 cm long, 22 cm wide, and 5.5 cm high. The total cost of the conditioner is currently less than $40 using components purchased at small-quantity retail prices. A detailed view is shown in Figure 3 and a list of materials, procured in the United States, is found in Table 1. All conditioners were constructed and tested for safety and functionality at PATH's product development facility in Seattle, Washington. Because of the high gas pressure required for operation of the cryotherapy device, there are safety concerns associated with introduction of an auxiliary device between the gas supply tank and the cryotherapy device. If inferior materials are used to construct the conditioner, prolonged blockage due to saturated desiccant or other mechanical blockage could lead to catastrophic failure of the conditioner.

**Table 1:** List of materials for the cryotherapy gas conditioner prototype. A list of materials for the cryotherapy gas conditioner prototype in which item numbers correspond to the parts in Figure 3. Manufacturer or distributor information and part number is included where possible.

**ITEM NO.**	**QUANTITY**	**DESCRIPTION**
1	1	HEX HEAD PLUG (1" NPT) [MCMASTER 4513K326]
2	1	COUPLING (1" NPT; 1.75" OD) [MCMASTER-CARR 4513K65]
3	1	SCHEDULE 80 1" × 3" NIPPLE [MCMASTER-CARR 7727K255]
4	1	TEE (1" NPT; 2.25" OD) [MCMASTER-CARR 4513K45]
5	1	SCHEDULE 80 1" × 6" NIPPLE [MCMASTER-CARR 7727K261]
6	1	90° ELBOW (1" NPT; 2.25" OD) [MCMASTER-CARR 4513K15]
7	2	SCOTCHBRITE PAD
8	2	ALUMINUM SCREEN
9	2	1" × 1/2" NPT HEX BUSHING [MCMASTER-CARR 4513K348]
10	2	1/2" × 1/4" NPT HEX BUSHING [MCMASTER-CARR 4513K344]
11	1	CGA 320 TO 1/4" NPT [WESTERN ENTERPRISES B-20]
12	2	FLAT TEFLON WASHER [WESTERN ENTERPRISES CO-6]
13	1	1/4" NPT NIPPLE [WESTERN ENTERPRISES CO-4]
14	1	CGA-320 NUT [WESTERN ENTERPRISES C0-2]
15	N/A	DRIERITE DESICCANT (NOT SHOWN)
16	N/A	TEFLON PIPE TAPE (NOT SHOWN)

### Survey of clinicians in Kenya and Peru

The planned evaluations of the prototype were considered by the PATH human subjects protection committee and deemed to be acceptable. After the initial bench evaluation at PATH, conditioners were distributed to three rural field clinics in Kenya using Wallach LL100 and Ascon MTC-810 cryotherapy units with N_2_O and one in Peru using the Wallach LL100 unit with CO_2_. Since all of these sites had reported problems with equipment blockage, it was our intention to assess whether providers felt that the use of the conditioners was associated with a decrease in the occurrence of cryotherapy equipment failure due to blockage. No systematic data were collected from these sites, only the general observations of the providers.

### Testing of the conditioner in Ghana

In order to quantify an effect that the conditioner might have on the ability to prevent equipment failure, we initiated a field study in May 2004 during a cryotherapy training session conducted by JHPIEGO in Ghana. During the training, one of the investigators (F.B.) performed mock cryotherapy treatments on beef sausage meat as surrogate tissue using one Wallach LL100 cryotherapy unit and beverage grade CO_2 _as the refrigerant gas. A fresh portion of meat sausage was frozen for periods of either 3 or 5 minutes and the time interval between consecutive freezing periods was varied from 5 to 20 minutes in 5-minute intervals. A non-randomized, comparative study on the prevalence of blockage of the cryotherapy device was conducted by alternating the use of the conditioner and the cough technique such that approximately one quarter of the freezes were done with the conditioner and without the cough technique, one quarter were done without the conditioner and with the cough technique, one quarter were done with both the conditioner and the cough technique, and one quarter were done with neither. The total number of simulated procedures was 101 and the order of times (3 or 5 minutes) and sequence of use of the four combinations of methods were performed in a non-random fashion. During the five-day evaluation period, F.B. alternately used the four different methods and recorded data on the ambient temperature, duration of freeze, time since the previous freeze, and occurrence of blockage.

Differences in the prevalence of cryotherapy failure due to blockage were compared using the chi-square test. A test for an interaction between the cough technique and conditioner in predicting blockage was done using binary logistic regression with both techniques and an interaction term in the model. Differences were considered statistically significant at p < 0.05. Analyses were done using SPSS version 12 (SPSS, Chicago, Illinois).

## Results

### Survey of clinicians in Kenya and Peru

Users of the conditioner devices in Kenya and Peru consistently reported that there were markedly fewer device failures due to blockage during cryotherapy while using the conditioner device. Providers who were using the Wallach LL100 cryotherapy unit also reported that prior to the use of the conditioner devices, their experiences were that the cough technique was not a reliable method to prevent blockage. There was a general impression that the time to achieve freezing of cervical tissue was reduced with the conditioner device.

### Testing of the conditioner in Ghana

Ambient evaluation temperature in Ghana varied between 36° and 39°C (97° and 102°F), representative of tropical clinic conditions. The anecdotal reports were from Kenya and Peru were consistent with the impression of the trainer (F.B.) during the experiments in Ghana. Additionally, he indicated that there was more ice crystal formation on the exterior of the cryotherapy device when the conditioner was not used. The data on the prevalence of blockage of the cryotherapy device under the four different conditions, using and not using the conditioner and the cough technique, are summarized in Table 2. The prevalence of cryotherapy device blockage during freezing was 0% (0/25; 95% confidence interval 0% to 13.7%)) with the conditioner alone, 23.3% (7/30; 95% CI 9.9% to 42.3%) with the cough technique alone, 5.9% (1/17; 95% CI 0.1% to 28.7%) with both the conditioner and the cough technique, and 55.2% (16/29; 95% CI 35.7% to 73.6%) with neither (p < 0.001). Mean durations of freezing periods attempted for the four conditions were comparable (Table 2).

**Table 2:** Data collected during simulated cryotherapy procedures in Ghana. Table represents the four conditions evaluated, the freeze duration, the time interval between freezing, and prevalence of blockage.

No cough technique or conditioner use				Conditioner use without cough			Cough technique without conditioner			Conditioner and cough technique		
Use sequence	Attempted freeze duration (minutes)	Time interval between freezing (minutes)	Blockage*	Attempted freeze duration (minutes)	Time interval between freezing (minutes)	Blockage*	Attempted freeze duration (minutes)	Time interval between freezing (minutes)	Blockage*	Attempted freeze duration (minutes)	Time interval between freezing (minutes)	Blockage*
1	3	10	N	3	15	N	3	Multiple days	N	5	5	N
2	3	5	Y	3	5	N	3	5	N	5	5	Y
3	5	15	Y	3	5	N	5	5	Y	3	5	N
4	3	10	N	3	5	N	3	10	N	3	5	N
5	3	5	Y	3	10	N	3	5	N	3	10	N
6	3	10	N	3	5	N	3	10	N	3	5	N
7	3	5	Y	3	10	N	3	5	Y	3	10	N
8	5	20	Y	3	5	N	5	720	Y	3	5	N
9	3	15	N	5	10	N	3	20	N	5	10	N
10	3	5	N	3	10	N	3	5	Y	3	15	N
11	3	10	N	3	5	N	3	10	N	3	5	N
12	3	5	Y	5	10	N	3	5	N	3	10	N
13	3	10	N	5	10	N	5	600	N	3	5	N
14	3	5	Y	3	10	N	5	10	Y	3	5	N
15	3	10	N	3	5	N	3	15	N	3	5	N
16	3	5	Y	3	10	N	3	10	N	3	5	N
17	3	10	N	3	5	N	3	5	N	3	5	N
18	3	5	Y	3	10	N	3	10	N	-	-	-
19	3	10	Y	3	5	N	3	5	N	-	-	-
20	3	10	Y	3	10	N	3	10	N	-	-	-
21	3	10	Y	3	5	N	3	5	N	-	-	-
22	3	10	Y	3	1440	N	5	660	N	-	-	-
23	3	10	Y	3	5	N	5	10	Y	-	-	-
24	3	5	N	3	5	N	3	10	N	-	-	-
25	3	5	Y	3	5	N	3	10	N	-	-	-
26	3	10	N	-	-	-	5	10	Y	-	-	-
27	3	10	N	-	-	-	3	5	N	-	-	-
28	3	5	Y	-	-	-	3	5	N	-	-	-
29	3	5	N	-	-	-	3	5	N	-	-	-
30	-	-	-	-	-	-	3	5	N	-	-	-
Mean freezing time: 3.14 min Failure prevalence: 16/29 (55.2%)				Mean freezing time: 3.25 min Failure prevalence: 0/25 (0%)			Mean freezing time: 3.48 min Failure prevalence: 7/30 (23.3%)			Mean freezing time: 3.35 min Failure prevalence: 1/17(5.9%)		

* Occurrence of blockage is denoted by Y, and lack of blockage by N.

**Figure 4:**
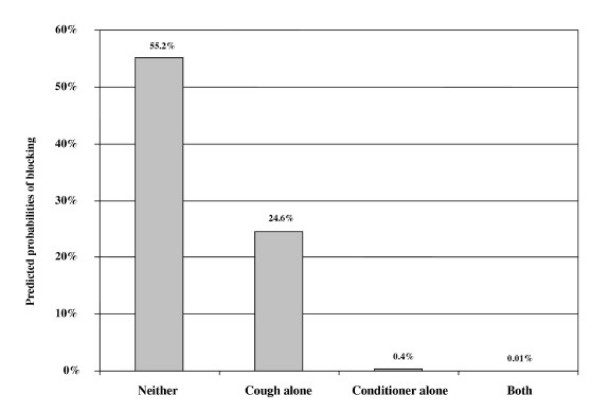
Predicted probabilities of cryotherapy equipment blockage. Predicted probabilities of cryotherapy equipment blockage with and without the cryotherapy conditioner, and with and without use of the "cough" technique from the logistic regression model.

In the binary logistic regression modeling, there was a statistically significant interaction effect (p < 0.05) suggesting that the cough technique was significantly improved when combined with the conditioner, but the main effect of conditioner alone was as strong as the combined effect. Predicted probabilities of blocking for the four combinations from the regression model are shown in Figure 4.

## Discussion

The use of the cryotherapy gas conditioner was associated with a dramatic decrease in the rate of cryotherapy device failure due to blockage, a rate reduction from 55.2% without the device to zero with the device, when testing was performed on non-human tissue. Because the exact reason for cryotherapy device blockage is unknown, we are unsure which of the proposed mechanisms of action are responsible for the beneficial effect of the gas conditioner. The high prevalence of failure during freezing without the conditioner indicates that, in this setting, use of a gas conditioner is preferred for consistency of cryotherapy service delivery.

We are aware of only one article that refers to the use of a similar device during cryotherapy procedures – in this case a commercially available gas purifier, used to improve consistency of cryotherapy operation during research on the topography and depth of cervical cryolesions [8]. In its cryotherapy training materials, JHPIEGO recognizes the existence of the problem in low-resource settings and describes the cough technique as a preventive measure for the problem of equipment blockage [5]. Our anecdotal and quantitative data show that the cough technique is only partially effective, reducing the rate of blockage from 55.2% to 23.3% during the mock procedures in Ghana. The predictive model shows that there is an interaction between the conditioner device and the cough technique, however the effect of the conditioner alone was so strong that it is unlikely that the combined use of the cough technique with the conditioner unit is justified.

Our study had several limitations. The non-randomized order in which the various combinations of conditioner unit and cough technique were used during the series of freezes and the close spacing between simulated treatments could have influenced the results in favor of the conditioner device to some extent. The freezing periods were often done in close succession which may increase the likelihood of device blockage. Sample size was not calculated a priori since there was not an estimate available as to the effect size of the gas conditioner. The only gas used in the Ghana study was beverage grade CO_2 _(an intermediate grade between industrial and medical grade) and it is not known if use of the more expensive, medical grade of gas would have averted the problem of blockage. The rates of cryotherapy failure in Kenya and Peru were not systematically measured before use of the conditioner, but instead the qualitative data were retrospective and anecdotal. We have not established supply lines, local cost of materials, or estimated times of delivery in countries outside of the U.S. The main strength of our evaluation was that the studies were conducted in three countries that experience problems with cryotherapy equipment blockage.

One of the impediments to systematic bench research on the problem of blockage has been the difference in failure rates in low-resource settings compared to the rates found either in developed-country clinics or in the laboratory. Failure at the bench can be difficult to induce and may not be entirely representative of the failure mechanism in a low-resource clinic (e.g., introduction of water into the supply gas line to mimic a hypothesized cause). Further research regarding the use of the conditioner should focus on four main areas. Confirmation is needed that, when the conditioner is used, an equal or lesser time is required to achieve an adequate ice ball on the cervix. Second, further evaluation of ice ball depth and temperature is warranted to ensure that the correct temperature and depth are reached or exceeded with the use of the conditioner. Third, the conditioner design could be refined to minimize weight and size while still maintaining safety and effectiveness. Lastly, it could be determined if the desiccant is necessary for adequate operation of the conditioner. Without desiccant, the conditioner could still provide an expansion chamber and filtering, lowering the overall cost and complexity of the device.

## Conclusion

This gas conditioning device shows potential to markedly reduce cryotherapy device failures and improve service delivery at clinics in low-resource settings that are experiencing this problem. Ideally, the conditioner will permit the clinician to employ a continuous freeze method, without the use of the cough technique, and provide adequate service despite the use of low-quality gas or difficult environmental conditions. The mechanism of beneficial action of the conditioner is hypothesized to be the reduction of moisture in the gas, removal of particulate matter, and elimination of liquid N_2_O or CO_2 _(via expansion and conversion to a gas) before it enters the cryotherapy device.

## Additional information

Complete plans for construction of the cryotherapy gas conditioner as presented in this article can be requested from the author.

## Competing interests

The author(s) declare that they have no competing interests.

## Authors' contributions

FB participated in the design of the field study, carried out the field study, and contributed to the manuscript. MH analyzed the data and contributed to the manuscript. JS and YS participated in the development of the device and the design of the field study, and contributed to the manuscript. All authors read and approved the final manuscript.

## Pre-publication history

The pre-publication history for this paper can be accessed here:


